# Medical Students’ Experiences With Virtual Reality Simulation Training: Qualitative Study

**DOI:** 10.2196/74301

**Published:** 2026-02-11

**Authors:** Vanshika Sharma, Sohee Park, Alexandra Voinescu, Chris Jacobs

**Affiliations:** 1 Department of Psychology University of Bath Bath United Kingdom

**Keywords:** health professions education, immersive technology, medical education, simulation, simulation-based education, usability, user experience, virtual reality

## Abstract

**Background:**

Beyond its applications in other settings, virtual reality (VR) technology has gained attention in medical education, offering immersive learning experiences. Previous research has demonstrated its potential as an educational tool in medical settings, highlighting enhanced educational outcomes, skill acquisition and retention, standardized training experiences, and the promotion of active learning. However, there is still a dearth of research exploring various aspects of VR user experiences, with most studies focusing on its effect on skill acquisition. Limited qualitative research further hinders an in-depth understanding of user experiences, restricting a comprehensive overview of VR’s potential in medical education.

**Objective:**

This study explored subjective experiences with VR simulation training and its perceived benefits and challenges among medical students in the United Kingdom, using the 5 domains of the Immersive Technology Evaluation Measure (ITEM).

**Methods:**

In July 2024, 15- to 20-minute in-person interviews were conducted with 11 medical students who had completed the immersive VR training consisting of the assessment and treatment of a virtual patient. Guided by the 5 domains of the ITEM as preconceived themes, a deductive thematic analysis was used to explore individual experiences with the training, embedded within narrative responses.

**Results:**

Findings aligned with the 5 a priori ITEM domains of system usability, immersion, motivation, cognitive load, and debriefing. Within these predefined domains, new subthemes emerged that enhanced the understanding of user experience. Participants reported usability barriers involving accessibility, technical issues, and limited variability in scenarios. Immersion was generally strong due to realistic environments, although reduced interactivity constrained authenticity. Motivation was reflected in active engagement and a greater sense of preparedness for clinical practice. Cognitive load was associated with divided attention, physical effects, and a need for clearer guidance and familiarization. Ultimately, participants valued debriefing sessions as valuable opportunities for reflection and reinforcing knowledge.

**Conclusions:**

VR training fosters immersion and motivation, but its effectiveness depends on balancing technical usability with cognitive demands. Future integration should prioritize design variability and structured debriefing to optimize learning outcomes. Refinement of immersive VR training in clinical education is also warranted, alongside further research in broader contexts and longitudinal use.

## Introduction

Virtual reality (VR) technology has emerged as a promising tool in various industries, including marketing [1], advertising [2,3], and education [4-6]. Using VR in health care, specifically in medical education, has been of growing interest in generating lifelike medical scenarios to provide students with more realistic and interactive learning experiences [7].

VR consists of computer-generated virtual environments experienced through various technical devices, including motion tracking, audio, and interactive hand controllers combined with head-mounted displays (HMDs) that generate 360° first-person perspectives of the scenario [8-10]. Such devices create a highly immersive virtual environment for users by offering real-time engagement in a realistic virtual environment, which extends beyond traditional simulations.

VR, with its immersive environment, can be highly beneficial in medical education. For instance, HMD-based simulations enhance ecological validity by mirroring the complexity and unpredictability of real-world clinical practice [9,11]. Learners in VR environments can be immersed in virtual hospitals, where they can explore interactive anatomical models, interact with patients, and practice surgical procedures more realistically [12,13]. Reflecting these benefits, systematic reviews have consistently highlighted that immersive VR-based medical simulations may be more effective than traditional methods across multiple outcomes, including clinical and soft skills, performance, self-efficacy, and stress and anxiety reduction [12-15].

Another benefit of this advanced technology is that it offers an immersive learning environment, allowing students to engage in highly realistic simulations of clinical settings without risking patient safety [16]. As such, VR simulations can increase confidence by reducing anxiety and stress and increasing self-efficacy [15,17]. Additionally, it allows students to make errors and derive lessons from them without encountering real-world repercussions, thereby cultivating confidence and competence [18]. In line with this, VR simulations have been found to improve surgeon safety behaviors through medical training in a risk-free setting, enhancing both understanding and adherence to safety protocols [19].

Numerous studies and meta-analyses have shown that VR training enhances both technical and nontechnical skills while fostering a positive learning environment. A systematic review by Zhao et al [20] reported that VR training significantly improved medical students’ clinical skills and performance compared to traditional techniques. Similarly, Harrington et al [21] found that VR training can match or even surpass conventional methods across a range of medical procedures and skills. Supporting this, Kyaw et al [22] conducted a systematic review and meta-analysis demonstrating that VR interventions improved posttraining knowledge and skills relative to traditional education. A further review comparing HMD simulations with traditional teaching formats (eg, slides, videos, 3D-printed or silicon models, and non-HMD simulators such as LapSim) showed that immersive VR accelerated the learning curve and increased motivation [12]. More recent umbrella reviews also reinforce these findings, highlighting that VR simulations improve learners’ skills, performance, and engagement [14,15].

Another potential advantage of VR in medical education is its ability to standardize training experiences, feedback, and assessment. Allowing for bias-free evaluations, providing real-time feedback, and enabling the recording of training data, it ensures consistent, reproducible training scenarios across learners, addressing the variability often encountered in traditional clinical training [15]. This standardization may contribute to more equitable learning opportunities and assessment practices while reducing the workload of instructors, as VR may require less supervision time [15]. For example, VR-based standardized patient encounters led to more consistent and objective assessments of medical students’ communication skills than traditional methods [23].

Despite initial cost concerns, advances in VR technology and affordable commercial HMDs may make training more accessible [24]. For example, a cost-effectiveness analysis by Farra et al [25] suggested that despite high initial costs, VR-based medical training programs may lead to significant cost savings over 3 years by reducing the need for physical resources and instructor time. Recent studies also indicated that simulation training may reduce the time required to achieve clinical competency and optimize training strategies [26].

User experience studies have recorded generally positive feedback on VR training; however, issues such as discomfort from the hardware or simulator sickness, a condition similar to motion sickness that occurs during or shortly after exposure to VR, characterized by symptoms such as nausea, dizziness, headache, disorientation, and eye strain, have been noted in several studies [11,12,15]. Nevertheless, recent research advancements have demonstrated promising results in mitigating simulator sickness and enhancing user comfort in VR systems intended for application across fields [27,28].

While existing research has substantially advanced the use of VR in medical training, much of the literature has primarily examined its role in skill acquisition and learning [15,29]. Comparatively less attention has been given to user experience–related factors, although a small number of studies have explored usability and technology adoption [30,31]. Moreover, the predominance of quantitative methodologies in this area [29] may further limit insight into the subjective and experiential aspects of VR engagement, which are not always fully captured through numerical measures alone. In line with this observation, a recent review highlights the relative scarcity of qualitative studies investigating the impact of VR on medical education [32].

Considering the limitations, this study used the domains of the Immersive Technology Evaluation Measure (ITEM) [33,34], a validated instrument, to comprehensively explore the user experience, precisely that of medical students, with VR technology in medical education. The ITEM was developed to capture the user experience of immersive technology in medical education [35]. The quantitative and qualitative content analyses of survey responses led to an online consensus meeting with key stakeholders, including doctors, allied health educators, technicians, and simulation administrators [33], on key aspects of measuring user experience, which guided the development of the 5 ITEM domains: immersion, motivation, cognitive load, system usability, and debriefing [33,34].

Grounded in the Model of Immersive Technology in Healthcare Education (MITHE), which explains multisensory experiences using interactions between technology interfaces, immersion, cognitive load, and motivational states [34], these domains serve as valid constructs for evaluating the effect of immersive technology on medical education. Furthermore, the domains were developed with expert consensus, capturing essential considerations for assessing user experience and ensuring relevance to real-world medical education settings. Integrating these domains, this research will address the limitations of prior studies by exploring underresearched user experience factors, which will provide insights into the potential of implementing VR technology in medical education.

Therefore, this qualitative study explores medical students’ experiences of VR simulation training, examining perceived benefits and challenges through the ITEM framework [33-35]. By capturing nuanced user perspectives, the study aims to inform how VR can be effectively integrated into medical education to support meaningful learning and more efficient training environments. Applying the ITEM framework to narrative data also enables further evaluation of its use in immersive medical education research.

## Methods

### Design

The study used a qualitative research design to explore the experiences of VR training among medical students with a deductive thematic lens, enabling the exploration of the use of the ITEM framework in understanding the experiences. Considering that a preexisting theoretical model from health care education guided our study, the postpositivist paradigm was considered most appropriate. Postpositivism recognizes the existence of an external reality while acknowledging that complete objectivity is unattainable, as observations are inevitably shaped by subjectivity [36,37]. The paradigm aligns with the deductive and theory-informed nature of our approach, while still recognizing the subjective dimensions inherent in experiential accounts.

### Procedure

Before the simulation, participants received a demonstration of using the VR devices in groups (4 students on day 1 and 7 students on day 2). Two teaching medical professionals were available throughout the simulation. They were registered medical practitioners with more than 2 years of teaching experience, working full-time as clinical educators for medical undergraduate education in the Department of Teaching at Great Western Hospital (GWH), Swindon, United Kingdom. Participants were instructed to undertake a medical consultation with a patient in VR using verbal prompts and to manage the randomly assigned clinical scenario. They were not informed of the diagnosis and had to assess the virtual patient to determine the diagnosis as if it were a real case. They completed the scenario using instructional materials embedded within the simulation. Teaching doctors and a virtual nurse provided clinical and technical assistance as needed. Each participant completed the simulation one after the other, while other members of the group were present. The simulation lasted between 20 and 30 minutes. The sessions took place over 2 consecutive days across 2 time slots.

After completing all scenarios, the 2 teaching medical professionals conducted a debrief with the entire group. This session lasted 10-15 minutes on average and discussed each student’s interactions with the simulation, along with the relevance of their clinical decisions to patient care and diagnosis. After the debrief, the study author (VS) conducted individual interviews with participants to explore their experiences with the VR training, which were recorded via Microsoft Teams [38]. Each interview lasted approximately 15-20 minutes.

### Participants

The study was conducted with medical students (n=11; 7 females and 4 males; age range 22-23 years) on clinical placement at the GWH. Participants were recruited from educational sessions led by the teaching medical professionals, and participation was voluntary. Participants opted to gain additional simulation experience via VR in identifying and managing sepsis, asthma, and anaphylaxis.

A purposive sampling strategy was used, wherein only students who had completed the VR simulation were invited to take part in the interview. The inclusion criteria specified that participants must be undergraduate medical students engaged in simulation-based teaching at GWH. For safety reasons, we excluded students living with epilepsy or other medical conditions that could be adversely affected by VR. Recruitment continued until no new themes were emerging from the data, indicating that theoretical saturation had been reached, resulting in a final sample size of 11 participants.

Students were informed that participation was entirely voluntary and could withdraw from participation at any time during the interview without providing a reason. They could also request the withdrawal of their data following participation until the data was fully anonymized.

### Materials

#### VR Simulations

**Figure 1:**
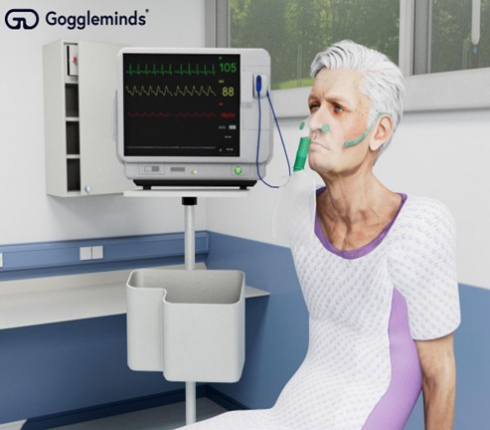
Virtual reality image of a patient presented to participants.

The VR clinical simulation used in the study was developed based on the prior simulation outlined in the case study by Jacobs et al [39] and instructional design principles. The simulation was created by Goggleminds and operated on a Meta Quest 2 HMD with 2 controllers. The virtual environments were designed to resemble acute medical settings in the United Kingdom, and the virtual patients exhibited typical characteristics of the illnesses presented (Figure 1). The room allowed freedom of movement in the simulated resuscitation room, which was approximately 4 m × 4 m in size and mapped to a UK hospital. Participants were ambulatory and could walk and touch objects within the environment using the controllers. The virtual room was equipped with necessary clinical instruments, such as a thermometer for measuring temperature and a mask for delivering oxygen.

The simulation incorporated 3 clinical scenarios (sepsis, asthma, and anaphylaxis). Each participant was randomly assigned to one of the scenarios and had to assess and treat the illness of the virtual patient. The clinical scenarios required participants to take a branched-decision patient history, perform clinical assessments, and medically manage the presenting illnesses in an appropriate manner. The clinical content was developed per national guidelines [40]. The VR sepsis training followed Merrill’s [41] 5 instructional principles aligned with Bloom’s taxonomy [42], providing an experiential, problem-centered simulation.

Simulation content and participant procedural steps were tested in a mannequin simulation environment prior to transfer of content to VR. Participants had access to a clinical history of the virtual patient at any point during the simulation by interacting with the virtual patient. Instructional materials were embedded in the simulation to guide participants through scenarios with a nonbranching logic sequence without logic operators or conditions for further action. A virtual nurse was present to execute participant commands, such as ordering tests or selecting treatments from a dropdown menu. Instructional cues and visual prompts guided decision-making, while embedded feedback and a facilitated debrief upon completion of the simulation supported reflection and consolidation of learning.

#### Semistructured Interview

A semistructured interview was conducted to explore medical students’ experiences of the VR training. The interview began with 3 background questions (job title, years of experience, and training duration), followed by 15 open-ended questions and prompts inviting participants to elaborate on their experiences. Selected questions were adapted from Swinnen et al [43], who used Bowen’s [44] framework for interview design, and refined to align with the ITEM framework and the specific context of VR in health care education. Additional questions were developed by the research team to capture feasibility considerations relevant to implementation (Multimedia Appendix 1).

Bowen’s [44] framework served as a complementary tool framing pragmatic aspects of feasibility, since it is particularly suited to assessing health care interventions that have already demonstrated effectiveness, shifting the feasibility to the phase of implementation. While Bowen’s [44] framework was reviewed to shape question development, the ITEM framework was adopted as the primary informing framework, serving as the conceptual foundation of the interview questions.

Grounded in the MITHE, ITEM provided a theory-driven structure for examining experiential, cognitive, and motivational aspects of immersive learning [34]. The final interview schedule was systematically reviewed to ensure alignment with the 5 ITEM domains (immersion, motivation, cognitive load, system usability, and debriefing) while incorporating relevant feasibility considerations derived from Bowen’s framework (Multimedia Appendix 1).

### Data Analysis

Interview transcripts were analyzed using deductive thematic analysis [45], guided by the 5 domains of the ITEM framework: immersion, motivation, cognitive load, system usability, and debriefing [33,34]. Predefined themes derived from ITEM structured the initial coding, while subthemes were allowed to emerge within each domain to capture nuances in participants’ experiences. This approach ensured that the analysis remained theoretically grounded while accommodating new insights from the data. The process was undertaken within a postpositivist paradigm, recognizing both the value of a structured, theory-informed framework and the interpretive role of the researchers in meaning-making.

Following the interviews, all transcripts were prepared verbatim and pseudonymized, and any identifying data were edited/redacted by VS before analysis, resulting in 11 transcripts. The thematic analysis followed the stages described by Braun and Clarke [46], including familiarization, code generation, code combination, theme review, determination of theme significance, and reporting.

Initial code generation was conducted using NVivo (version 12; Lumivero) [47] by 2 study authors (VS and SP) who independently reviewed the transcripts and reached consensus on the codes. As the study used preconceived themes, codes were categorized under the relevant ITEM domains, with subthemes generated as necessary. Subsequent procedures of the analysis were conducted by VS, SP, and the other 2 authors (AV and CJ) to generate the final 5 themes, 10 subthemes, and 23 codes through researcher consensus.

### Ethical Considerations

Ethical approval for the study was obtained from the University of Bath (Data & Digital REC; reference 5416-5731). Following the University of Bath's Research Data policy, fully anonymised data was stored on university-managed servers and temporarily on a password-protected, encrypted personal computer. Only the researchers had access to the data during the study. Informed consent forms and fully anonymised data will be stored for at least 10 years in the University of Bath Research Data Archive. Participants were provided with a brief study overview in the advertising email. They received a detailed information sheet and provided written consent confirming their understanding of participation before proceeding with the study. All participants received £10 (US $13.69) as compensation for their time and participation in the study.

## Results

### Overview

**Figure 2:**
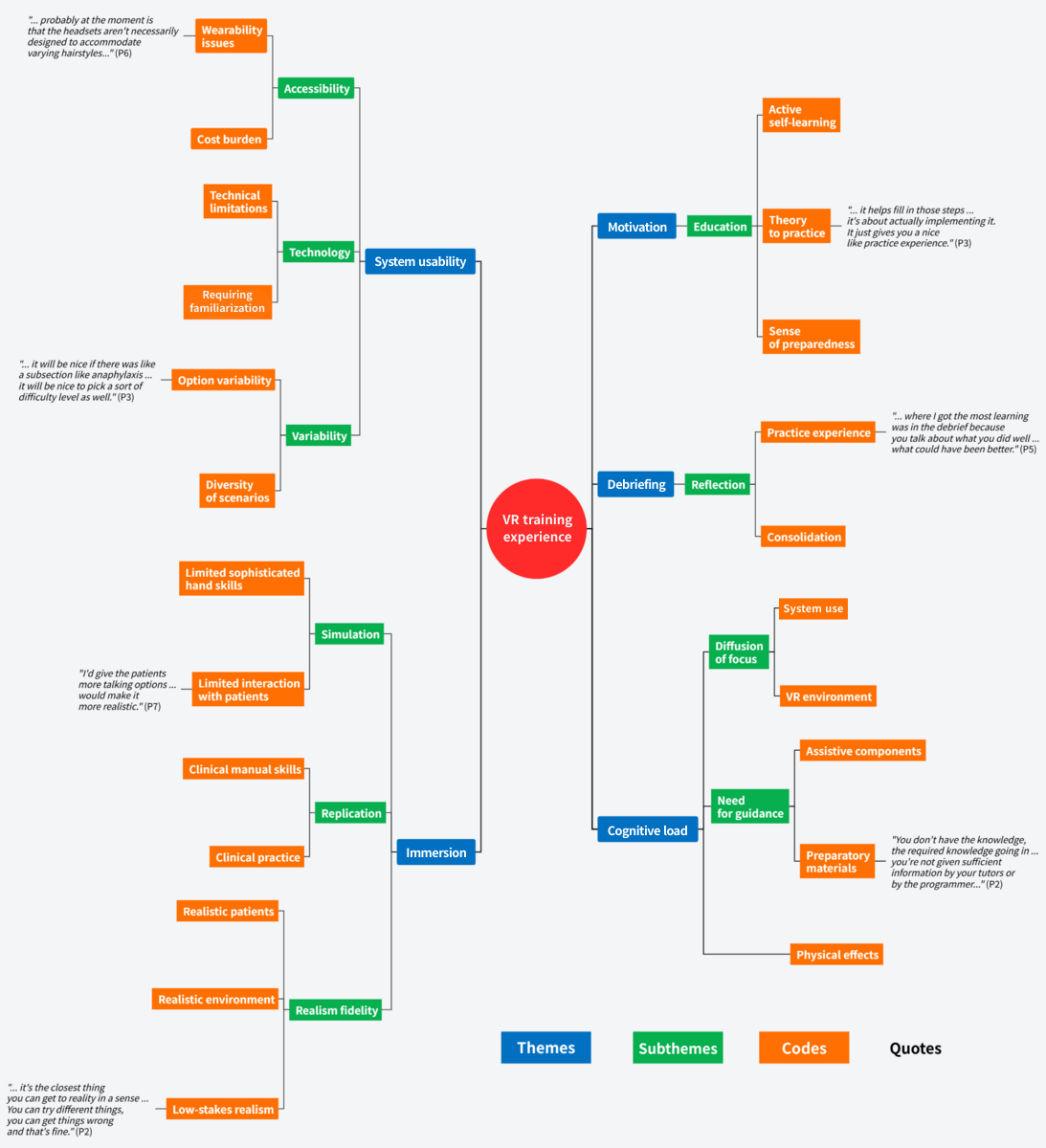
Thematic map with themes, subthemes, codes, and exemplary quotes. VR: virtual reality.

**Figure 3:**
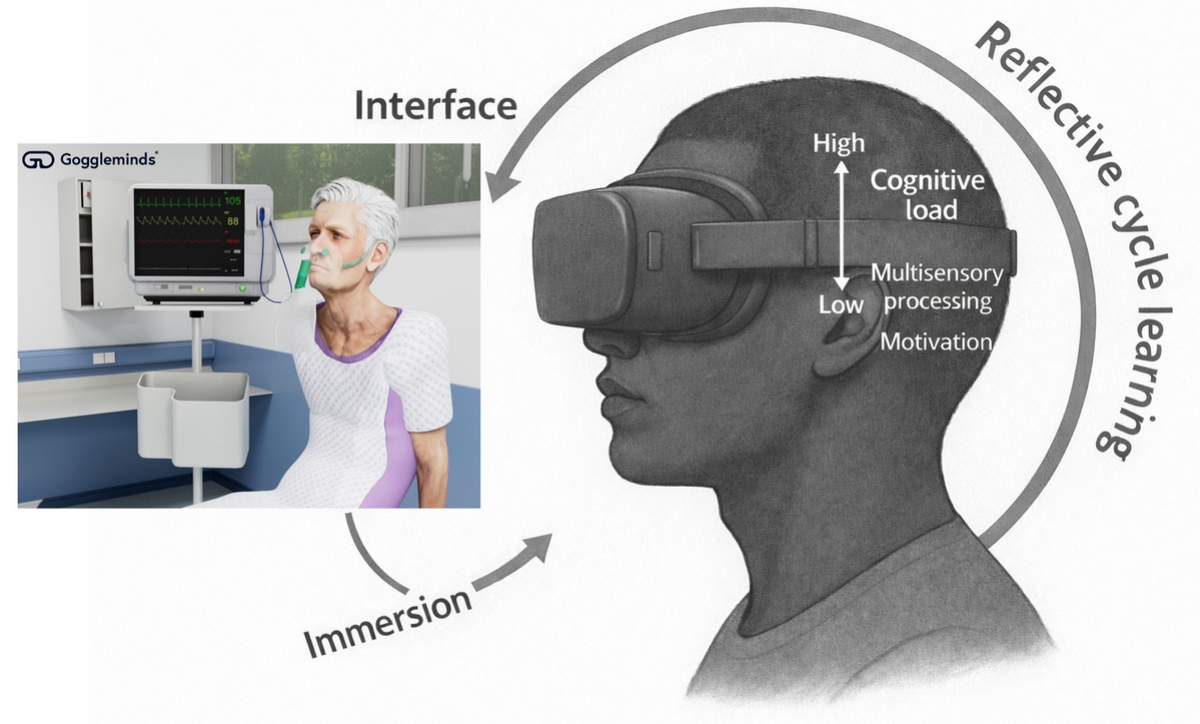
Adapted version of the Model of Immersive Technology in Healthcare Education.

The analysis of medical students’ experiences with VR training was structured around the 5 domains of the ITEM framework: immersion, motivation, cognitive load, system usability, and debriefing. These domains highlighted both the strengths and limitations of VR as a training tool (Figure 2). Within each domain, subthemes were developed from participant narratives, resulting in 5 overarching themes, 10 subthemes, and 23 codes agreed upon through researcher consensus. The findings illustrated the interrelated nature of the ITEM domains, showing how immersive, motivational, cognitive, and usability factors interacted to shape learning experiences. This dynamic interaction reflected the broader mechanisms described in MITHE [34], where multisensory engagement and iterative practice underpin deeper understanding and skill refinement (Figure 3).

### System Usability

System usability covers the issues around the VR training that could limit its efficient usage. The theme covers 3 different issues, denoted as subthemes: accessibility, technology, and variability. Accessibility covers how the physical discomfort of the headset and the cost of training can limit its access. Technology includes the technical limitations of this design and the need for more time for familiarization. Finally, variability reflects the need for having diverse scenarios and levels of difficulty for practice.

Participants raised some concerns regarding the usability of the VR system. One key issue was the wearability of the headsets, which reduced the accessibility of the training. One participant explained, “I wear glasses, and they usually cause a little indent on my nose because of the pressure” (P6). Another participant noted, “It’s a relief to take it off after a session” (P3), indicating that the physical discomfort limited the engagement with the system. Additionally, the perceived cost of implementing VR training was identified as a barrier to its ubiquitous use in medical education (P5).

Participants also encountered minor technical limitations like limited test options and occasional incorrect labels that disrupted their learning. One participant described the frustration caused by software issues, stating, “The bugs and glitches meant that certain options weren’t available to us” (P2). "Another raised the faults within the simulation, remarking, “So for example, like I think this was like the kind of glitch of like getting a medication and the nurse being like this isn’t needed right now, and it kind of is the correct thing to do ... so often things just don’t quite marry up" (P10).

Some suggestions for improving system usability also centered on familiarization. Participants voiced the difficulties faced in using the unfamiliar VR system and recommended allocating time for familiarization. One participant noted, “... but it was quite a lot of information to take on ... it would be helpful to have some kind of extra time outside of the scenario to familiarise ourselves ...” (P11).

The need for more significant variability in the VR training was highlighted across participants. One key issue was the lack of diversity in these training resources, which some participants considered a space for improvement. One participant noted, “It will be nice if there was like a subsection like anaphylaxis ... it will be nice to pick a sort of difficulty level ...” (P9), underscoring the importance of variability in options and levels of difficulty in each scenario. Additionally, participants expressed a desire for scenarios beyond the standard scenarios they typically train for in medical training (P3).

### Immersion

Immersion emerged as another central theme, comprising simulation, replication, and realism fidelity. Simulation highlights limitations in practicing hands-on skills and patient interaction within the simulation due to language model constraints. In contrast, replication includes a positive experience of the rehearsal of some manual skills and clinical routines, while realism fidelity reflects the consensus for providing authentic patient scenarios, realistic clinical environments, and a safe, low-stakes context for realistic training experience.

Participants frequently highlighted the realistic clinical environments they encountered through the sensory inputs in the VR training. Participants remarked, “... you can listen with a stethoscope and there are actually heart sounds ... it does do a good job of simulating real life practise ...” (P5), “I quite liked that everything was a bit intuitive, so the room was like small, but it was representative of the normal hospital room there” (P3), illustrating how the VR training effectively incorporated visual and auditory aspects in actual clinical settings into the VR environment to enhance realism. Additionally, participants noted that the virtual patients closely resembled real-life patients with the illnesses presented in each scenario (P5). Furthermore, participants valued the opportunity to engage in realistic practices without safety risks, which encouraged them to explore a broader range of patient assessments and management techniques (P2).

Many participants praised VR for its high-level replication of clinical practice overall. One participant commented, “... it was a bit more realistic in the sense of having to turn the oxygen on ... rather than everything being done with one click” (P11). This suggests that VR training realistically mimics step-by-step clinical procedures, enhancing the immersive experience. Additionally, participants frequently acknowledged the benefit of the VR simulation for requiring manual skills needed in medical practice. For instance, one participant noted, “You can actually have to grab things and apply them ... say the port of simulator and put it on the patient” (P6), reflecting how the training facilitated the exertion of manual skills that medical professionals ought to be equipped with in real life.

However, some noted limitations in realism within simulation. Some participants reported limited tactile realism in simulation, claiming that the training did not allow them to practice practical skills involving direct and more sophisticated manual interaction (P5). Additionally, participants identified a lack of realism in the training due to limited patient interaction. One suggestion for improvement was to enhance communication options in VR, as noted by a participant: “I’d give the patients more talking options. I think in clinical practice, one thing that is highlighted to us is the communication with our patient ...” (P7). Addressing this in VR training could better prepare medical students for effective patient interactions.

### Motivation

Motivation reflects the actuating role of the training with education as a subtheme. Education as a subtheme highlights how VR training fostered medical students to actively engage in learning independently through simulated clinical scenarios and to apply theory to practice during the training. The sense of preparedness denotes how the training helped them prepare for practicing their skills in real-life scenarios, providing a sense of self-assurance.

VR was praised for encouraging active learning, requiring them to actively engage in decision-making and problem-solving on their own during clinical scenarios. One participant explained, “But as long as the person who’s training you stays quiet ... It forces that decision-making process ... as opposed to studying from a textbook or notes where learning is more passive” (P5). This self-directed and active engagement was a significant advantage, helping translate theoretical knowledge into practice. One participant reflected, “VR training helps me think step-by-step about things like how to prescribe fluids or who to ask for help with bloods” (P3). Hence, self-efficacy and agency are promoted as motivations to drive learning.

Another key outcome of the training was an increased sense of preparedness as future medical professionals. Several participants noted that practicing clinical protocols in the virtual environment enhanced their preparedness for real-life scenarios. One remarked, “It made me more confident about running through a protocol in the moment” (P4). This immersive experience was perceived as a valuable tool for learning, fostering confidence, and improving clinical skill retention.

### Cognitive Load

Cognitive load reflects both the inherent cognitive demands of completing the clinical task (ie, intrinsic load) and the additional effort created by navigating the VR system (ie, extraneous load), which for some students contributed to discomfort and divided attention. Two subthemes emerged: diffusion of focus and prior guidance. The theme also includes some physical effects experienced by the participants, which may have surfaced due to the cognitive discomfort, such as motion sickness. Diffusion of focus as a subtheme includes the reported displacement of focus from the training to using the VR system itself. Finally, prior guidance includes the need for training and orientation to be better prepared to use the technology and the inclusion of assistive components in the system to better navigate the training.

Participants experienced varying cognitive load during the training, as reported by physical impacts. A notable issue was motion sickness, with a few participants reporting discomfort. One participant shared, “I had a lot of times where I was zooming in and out, which made me really dizzy” (P1).

In addition to physical discomfort, many found that using the system and navigating the VR environment (P5) created a cognitive burden, distracting them from clinical tasks. One participant noted, “Half my focus was actually on figuring out how the system works” (P1). This challenge, coupled with the need for prior knowledge to use the VR training effectively, left some participants feeling unprepared for the sessions. One respondent mentioned, “If you don’t have the knowledge going in ... it feels like a waste of time” (P2), implying that improved preparatory materials or instructions could alleviate the cognitive burden and enhance their learning experience.

Additionally, the need for training assistance in the system was suggested, as noted by one participant: “... maybe something like voice recognition ... like maybe some sort of assistant thing could pop up” (P9), reiterating that additional assistive support before and even during the simulation could benefit medical students in VR training*.*

### Debriefing

Debrief emerged as a central theme, with reflection identified as a key subtheme. Reflection encompassed the positive aspects of both the critical review of the training session and the consolidation of learning, underscoring its importance in reinforcing clinical understanding and supporting the translation of experience into improved practice.

Across the board, participants regarded the debriefing sessions following VR training as one of the most beneficial aspects of the program. These sessions allowed participants to reflect on their experiences, discuss mistakes, and consolidate their learning. One participant stated, “What helps consolidate the memory is the debrief after the session ... I learn the most when I’m able to practice and make mistakes” (P7). Another echoed this, noting, “Where I got the most learning was in the debrief” (P5). This feedback indicates that debriefing is crucial in reinforcing the learning outcomes of the VR training by reflecting on the experiences and consolidating the knowledge gained.

### Summary

The results indicate that VR training offers significant benefits, including immersive learning experiences, enhanced motivation, and reflection through debriefing. Acknowledging the benefits, participants also identified areas for improvement, such as expanding communication options and enhancing tactile realism to facilitate more immersive learning. Additionally, they emphasized the need to reduce physical and cognitive burdens associated with the training. The findings also highlight the importance of improving system usability and training variability while addressing technical and headset wearability concerns.

## Discussion

### Overview

Despite extensive research on the implementation of VR in medical education, few studies have explored a broad range of user experience factors. Moreover, prior research has predominantly relied on quantitative methods, limiting a comprehensive understanding of participants’ experiences with VR. This study begins to narrow these gaps by qualitatively exploring medical students’ VR training experiences, using semistructured interviews and deductive thematic analysis [45] to provide in-depth insights beyond numerical data. Guided by the 5 domains of the ITEM [33,34], the study aligned with the 5 themes, 10 subthemes, and 23 codes to explore broader key constructs for assessing VR in medical education. Adopting a postpositivist, theory-informed perspective, the discussion interprets these findings in relation to established models of immersive learning, highlighting how the ITEM domains interact and reflect the experiences of potential users (ie, medical students).

### System Usability: Technical and Psychological Dimensions

The participants frequently highlighted usability challenges, especially those pertaining to wearability. Students wearing glasses reported discomfort due to headset pressure, limiting engagement, reflecting on findings by Kardong-Edgren et al [48] on similar usability issues in VR medical training. Additionally, although participants found VR training immersive and educationally valuable, they reported concerns regarding its costs. The participants reported that the cost of implementation would hinder the ubiquitous use of VR training, which aligns with a review that emphasized the need to address cost issues of VR in medical education [29].

Technical limitations disrupted the VR training in some instances, which participants often reported to impact their experiences with the training. For example, there were instances where the VR environment froze for a few seconds, the sound stopped, or the headset was out of order, which may have negatively affected user experience. Although such limitations were resolved during the training, such as by restarting the VR environment and the headset, these emphasize the importance of usability and comfort in VR design, advocating hardware adjustments to improve user experience [49].

These technical limitations not only affected usability but also undermined participants’ sense of autonomy and competence, which are critical elements in motivation for learning. Past research has identified presence and immersion as key factors that positively influence experience in VR, and are associated with perceived autonomy and competence [50]. For instance, presence and immersion may enhance the sense of autonomy by increasing participants’ perceived control over their virtual behaviors and decision-making [51]. Similarly, users’ perceived competence may increase with greater presence and immersion in the simulation, particularly through seamless interactions with the virtual environment [51]. As a result, the intermittent pause of the training sessions to resolve technical issues may potentially have disrupted the immersion and sense of presence in VR of participants, reducing the sense of autonomy and competence. Our findings indicate that system usability is crucial not only for ease of use but also for enhancing learners’ immersion and presence, which in turn support their sense of autonomy and competence during training.

Participants frequently expressed the need for variability within the VR training. Specifically, they emphasized the need for a broader range of scenarios in terms of diseases, as well as options for treatment embedded within each scenario. Although participants appreciated the realistic clinical scenarios relevant to their future practice, they suggested expanding the range to include a wider variety of diseases. Additionally, participants suggested including drug subsections when selecting treatment options in the simulation, along with adjustable difficulty levels to enhance comprehensiveness and applicability. Recent evidence from educational VR studies [52-54] supports that flexible scenario difficulty and customization enhance motivation for learning by reinforcing competence and autonomy. This highlights the importance of variability in VR medical training to enhance motivation for learning, ultimately enhancing learning outcomes.

The need for sufficient time to familiarize oneself with VR technology was a recurring theme. Many participants initially felt overwhelmed by the VR setup process, including using the controllers and navigating the scenarios within the environment. They were concerned that learning the VR setup might disrupt their focus on the clinical tasks and suggested a dedicated preparatory period to allow sufficient time for familiarization. Such remarks are aligned with the findings by Ghanbarzadeh and Ghapanchi [55], which highlight that familiarity with VR platforms reduces the learning curve, allowing students to engage more effectively with clinical tasks. Findings by Jung and Lee [56] support this approach, recommending dedicated VR practice sessions to help participants familiarize themselves with the environment and controllers, thereby enhancing their sense of presence and better preparing them for clinical practice.

### Immersion and Cognitive Processing

The interviews revealed that participants appreciated the high fidelity of the VR training, emphasizing its close resemblance to real clinical environments. They appreciated the realistic visual representation of the clinical apparatus, patients, and auditory elements, including heart sounds, aligning with prior research demonstrating that greater realism fidelity enhances immersion [57]. Participants also acknowledged that the training successfully replicated clinical practice, incorporating patient assessment and equipment selection within the scenarios. The inclusion of manual tasks, such as requiring them to handle simulators and adjusting oxygen levels using controllers, further anchored users in the clinical settings, showing high resemblance to real-life clinical practice. These narratives are consistent with a study that demonstrated the importance of active participation, including administering diagnostic assessments and therapeutic measures using procedural skills, to strengthen the sense of presence and immersion [58].

Participants also valued the opportunity for low-stakes clinical practice through the training, enabling repetitive training of clinical skills and clinical procedures without safety risks. This aligns with the concept of deliberate practice introduced by Ericsson et al [59], a method shown to improve performance in medical education by providing ample opportunities for gradual refinement of learner performance [60]. For learners to achieve deliberate practice, engagement in focused and repetitive practice of skills is essential [61,62], which is often limited in real clinical settings [60,63] due to safety risks. In this context, immersive VR medical training serves as a platform for clinicians to repeatedly practice skills in a risk-free environment and further analyze their performances for skill improvement. Thus, the VR training is an effective tool for medical students to achieve deliberate practice [16], and is anticipated to enhance their performances in future clinical settings, driving the benefits demonstrated in the previous studies [15-19].

Despite these strengths, some participants highlighted limitations that diminished realism. While the training included manual tasks, participants reported limitations in performing practical and sophisticated skills using their hands because the simulation required using controllers to complete manual tasks, restricting haptic feedback and natural hand movements. In the medical context, studies have shown that haptic feedback enhances realism by providing tactile sensations, thereby making VR training more immersive [64]. Additionally, research indicates that allowing users to perform object-specific natural hand gestures improves immersion in VR training [65]. Despite the importance, realistic haptic feedback is still viewed as a major missing piece [66], and naturalistic hand-object interaction is still limited [67], which may considerably diminish the degree of immersion. The narratives, therefore, illustrate the limitations of current VR technologies, calling for the incorporation of these features to include precise manual tasks, which would enhance realism.

Another limitation noted was the lack of patient interaction, including limited verbal exchanges and opportunities for history-taking. Patient interaction is critical to effective health care delivery and improves therapeutic outcomes and well-being [68,69]. Additionally, simulations with interactive virtual patients enhance realism and foster essential clinical reasoning and history-taking skills [70]. These underscore that sufficient training is needed for medical students for effective patient interaction in actual clinical settings. Therefore, incorporating interactive features into VR training could enhance immersion and prepare medical students for real-world patient interactions, enhancing training outcomes.

### Motivation and Self-Determination Theory

Participants acknowledged the VR training for its ability to foster self-directed and active learning. They described the training as a more engaging alternative to traditional text-based education, requiring independent decision-making and systematic thinking. This is consistent with studies showing the efficacy of VR-based training in promoting self-directed and active learning in simulated environments [18,71]. Participants also experienced self-directed and active learning through applying theoretical knowledge to practice, remarking that the training encouraged them to consider treatment steps systematically based on their theoretical knowledge. This aligns with the previous findings, highlighting that VR training fosters active and independent learning accomplished by applying theoretical knowledge in practice, which is crucial for medical education [72].

Self-determination theory (SDT) [73], a human motivational theory that focuses on understanding autonomy functioning, provides a valuable framework for understanding these findings, specifically in the context of VR-based learning. According to SDT, intrinsic motivation leads to high academic performance, which has also been demonstrated in medical education [73-75]. According to the theory, maintaining and enhancing motivation requires satisfying 3 basic needs: autonomy, competence, and relatedness [73]. Autonomy refers to an individual’s perceived control over their actions [76], competence refers to the ability to effectively interact with the environment and the confidence to achieve desired outcomes [77], and relatedness refers to the need to form meaningful connections and interact with others during an experience [78].

From an SDT perspective, the VR training supported autonomy by allowing participants to engage in self-directed learning and independently apply theoretical knowledge to practical scenarios, actively helping them to have control over their actions. Additionally, it is possible that participants achieved competence through interacting with realistic patients and a clinical environment to some extent, taking actions to achieve desired clinical outcomes. Therefore, the narratives demonstrate the potential of the VR training as a tool for fostering motivation in medical students if technical optimization is entailed.

The participants’ sense of autonomy in SDT is also closely linked to a sense of agency in VR, defined as the user’s perception of control and ability to influence their virtual environment and its consequences [79]. The VR training allowed medical students to interact with virtual medical equipment and make step-by-step independent decisions in simulated scenarios. These components are likely to increase a sense of agency, which is crucial for promoting learners’ autonomy and self-directed learning in VR environments [80,81]. Thus, VR training that provides a high sense of agency likely supports students’ autonomy, enhances motivation, and improves learning outcomes [73,82].

However, given that the technological limitations potentially diminished the sense of presence and immersion, this motivational triad may have been vulnerable to disruption. Technical limitations, headset discomfort, or confusing interface design that were remarked by participants might have directly undermined the sense of immersion and presence, leading to diminished feelings of competence and autonomy [50,51], thereby diminishing motivation. Participants who struggled to use the system effectively expressed frustration and dependency, an example of how usability issues can thwart psychological needs and reduce motivation even in otherwise engaging environments. Similar findings in immersive learning research show that poor usability elevates extraneous cognitive load and negates the benefits of motivation [83]. Hence, motivation in VR environments is contingent on technological reliability as much as on instructional quality, calling for technological optimization in VR training to foster motivation for learning.

Although they may have experienced limited motivation, participants highlighted that the training provided a sense of preparedness, giving them a sense of readiness to apply these skills in real-life contexts. Among medical students, active learning sessions and practical applications have been shown to help students feel a sense of preparedness, boosting confidence levels [84]. This may indicate that the structure of the training that requires active learning and provides opportunities to apply their knowledge into practice successfully helped students to prepare for future clinical practice, highlighting the importance of incorporating such components in medical education using VR.

### Cognitive Load and Managing Complexity

Participants frequently described VR training as cognitively demanding, particularly when navigating virtual environments. Dedicated attention to the interface and VR environment interrupted the full concentration on the clinical tasks and created additional cognitive strain, echoing prior findings that immersive learning environments may inadvertently elevate extraneous cognitive load [85]. Physical effects, including motion sickness and visual fatigue, further compounded this burden, reflecting how physical discomfort can interfere with concentration [86]. While some cognitive challenge is pedagogically beneficial, excessive demand risks overshadowing core learning objectives. As seen in the narratives of participants, providing assistive components embedded within the simulation and preparatory materials could reduce unnecessary load and enable learners to focus on clinical reasoning. This is consistent with the recommendations of Howard and Lee [87], who emphasize attentional guidance and pretraining interventions to balance complexity and cognitive efficiency.

Crucially, from the narratives, system usability emerged as a core determinant of cognitive load. Participants who struggled with headset wearability or technological issues reported cognitive strain and distraction, suggesting that design flaws amplify mental workload. Thus, usability and cognitive load should be considered reciprocal: improved interface design directly alleviates cognitive demands, while excessive mental effort can in turn exacerbate perceived usability issues. This relationship reinforces that technological design and cognitive design are inseparable aspects of effective simulation pedagogy.

Reported cognitive load can also be examined through the lens of realism and fidelity. According to cognitive load theory [88], all novel information is primarily processed by working memory, which is limited in capacity and duration. Therefore, in virtual environments that provide high immersion, users may encounter a flood of new information, increasing their cognitive load [89]. For instance, immersive VR was found to increase cognitive load and decrease cognitive engagement [90], which may impact learning. Additionally, immersive VR was perceived as more distracting, potentially due to the increased cognitive load [91]. From these findings, it can be inferred that high immersion may enhance the sense of agency and competence in learners, leading to increased motivation. However, it can simultaneously increase cognitive load when learners allocate too many attentional resources to managing the interface rather than to clinical reasoning. Given that a familiarization period reduces cognitive load in VR users [89], the potential increase in cognitive load from high-fidelity VR can be alleviated by providing the preparatory materials suggested by participants. Additionally, this highlights the need to balance fidelity with the cognitive resources required to optimize training and promote positive learning outcomes.

### Debriefing to Integrate Reflection and Relatedness

Debriefing emerged as a critical process through which learners transformed immersive experiences into lasting knowledge. Participants consistently described postsimulation discussions as where “the most learning happened,” providing practice experience. Debriefing allowed errors to be reframed as opportunities for growth, consolidating procedural and conceptual understanding. These findings align with Fanning and Gaba [92] and McGaghie et al [93], who identified structured debriefing as essential to simulation-based learning. From an SDT perspective, debriefing fulfilled the need for relatedness by creating a supportive environment where learners receive feedback, validation, and social connection, as factors strongly associated with sustained motivation [94-96]. Additionally, debriefing serves as a crucial component for improving performance through deliberate practice. [59]. According to Ericsson et al [59], debriefing enabled learners to gradually refine and improve their performance through immediate expert feedback after training, providing opportunities for reflection and correction of choices, and ultimately facilitating deliberate practice [62].

In this sense, debriefing served a restorative function within the ITEM system. Where cognitive load or usability barriers temporarily undermined autonomy, competence, and confidence, debriefing reestablished equilibrium, reinforcing learning, and reengaging motivation. This balancing role highlights why debriefing should not be considered an adjunct but a core pedagogical domain within immersive education. Additionally, the feedback from medical practitioners during debriefing further enabled learners to reflect on their performance, which is essential for the improvement of performance and skills. Overall, narratives highlight the importance of debriefing in medical education to facilitate motivation and performance improvement essential for future clinical practice.

### Synthesis and Educational Implications

Overall, the findings indicate that immersive VR training involves a complex interaction between immersion, usability, cognitive load, motivation, and debriefing. High immersion may enhance realism and engagement, but without sufficient usability and scaffolding, it can contribute to cognitive overload. Similarly, motivation, which is crucial for learning, depends on the fulfillment of autonomy and competence, which may be disrupted by technical difficulties or disorientation. Debriefing appears to function as a recalibrating mechanism, supporting these psychological needs through reflection and feedback. Additionally, its role as an opportunity to receive constructive feedback and reflect on performance further highlights the importance of debriefing for performance improvement and enhanced motivation. The educational value of VR, therefore, arises not from any individual domain but from the integration and balance among them.

Moreover, such a comprehensive interpretation of narratives emphasizes the importance of balancing technological and psychological fidelity, which would potentially guide future VR simulations. According to the narratives, supporting autonomy (through user control), competence (through graduated difficulty and responsive feedback), and relatedness (through debriefing and collaboration) can sustain engagement and deepen learning, while cognitive load should be managed for optimal learning. To achieve this, future VR simulations can incorporate prebriefing, intuitive interfaces, adaptive difficulty levels, variability in scenarios, and ergonomic hardware to reduce extraneous load. Equally, structured debriefing should remain a central component to reinforce reflection, motivation, and further skill improvement. Ultimately, the goal is not maximal immersion but optimal integration, where cognitive, affective, and technological dimensions align to create meaningful, sustainable learning experiences.

### Limitations

While this research offers valuable insights into VR adoption in medical education, some limitations are noted. Participants only underwent the training once before being interviewed without further engagement in the training. This may not have accurately reflected the experience of regular VR use in training over time, which potentially limits our understanding of implementing the technology in medical education. The retrospective nature of the responses also could have introduced information bias due to potential memory inaccuracies. Additionally, the ITEM framework used for data analysis might have restricted the focus to responses within its scope, suggesting the use of an inductive approach for future exploration.

The study’s scope was limited to medical students from one UK hospital. This qualitative research aimed to gain in-depth insights into the experiences and perceptions of a specific group of VR training users; thus, the results cannot be generalized to other professions. Future research may encompass a broader population, including various job roles, expertise levels, academic institutions, and geographical locations, to provide a more comprehensive understanding of perceptions.

### Conclusions

This qualitative study examined medical students’ subjective experiences with VR training. Participants valued the immersive learning experience, highlighting its role in fostering motivation and providing effective debriefing. However, areas for improvement emerged, including the need to reduce cognitive load, enhance realism, and improve system usability. The findings suggest that the effectiveness of VR-based learning depends on balancing these domains rather than maximizing any single aspect. Incorporating these findings could guide advancements in VR as a reliable tool for medical education. Future research should use longitudinal designs and include diverse populations to broaden understanding of VR’s effectiveness in medical education, which can help determine whether initial perceptions of excitement or apprehension, potentially influenced by the novelty effect of VR, are sustained, diminished, or evolve with repeated use. Such research would also provide insight into the long-term educational impact of VR training, including knowledge retention, transfer of skills to clinical practice, and the durability of motivational and immersive benefits. By aligning technological fidelity with psychological and pedagogical principles, immersive VR can evolve into a sustainable and effective component of medical training.

## Appendix

Multimedia Appendix 1 Interview schedule and its alignment with the frameworks (table and map format).

## Abbreviations

GWH: Great Western Hospital
HMD: head-mounted display
ITEM: Immersive Technology Evaluation Measure
MITHE: Model of Immersive Technology in Healthcare Education
SDT: self-determination theory
VR: virtual reality
