# Supplementary material for: Medical Students’ Experiences With Virtual Reality Simulation Training: Qualitative Study
Source: JMIR Med Educ. 2026 Feb 11;12:e74301. doi: 10.2196/74301 (PMC12936657; doi:10.2196/74301)

**[Question list]**

The questions below are listed in order they were presented to participants. Numbered questions denote the main questions, and indented questions denote prompts that were used when necessary. Baseline questions were asked to collect participants’ demographics and general questions were asked to explore their initial perceptions and experiences with VR technology in general.

(Baseline) Can you please say your job title?

(Baseline) The length of time working in this role?

(Baseline) For how long have you been training?

(General question) In general, what do you think about VR technology?

(General question) Do you have any experience with it?

1. What do you remember most about the VR training?

Why?

2. Do you think the VR training has helped you achieve your learning objectives?

If so, how exactly did it help you?

Which part helped you the most?

3. Would you like to continue the VR training?

4. What adjustments would be necessary to make the training optimal for you?

5. Did you find the VR training useful?

If so, what benefits did you feel?

6. Would you recommend the training to your colleagues?

7. What are in your opinion the main challenges to the use of VR in medical training?

8. How did you feel during the VR training?

9. Did you enjoy it?

What part did you like the most?

10. In your experience, what might go wrong with this VR training?

11. What would you do to make it work better?

12. Can you think of some characteristics of VR technology that can help with engaging with it?

13. Do you have any concerns or safety issues about this VR training?

14. Are there any barriers you can think of that can impact the uptake of this VR training on a larger scale?

15. Do you have any questions or would like to raise any other issues regarding the topic of discussion that were addressed in the interview?

**[Interview guide table]**

The table below illustrates how each question, except for baseline and general questions, align with the two frameworks and their domains. “Notes on alignment” further describes how the questions were designed to align with the selected domains.

| Interview Question | ITEM Domain(s) | Bowen’s Feasibility Domain(s) | Notes on Alignment |
| --- | --- | --- | --- |
| 1. What do you remember most about VR training?  - Why? | Immersion; Intrinsic Motivation | Acceptability | Captures the recall of salient immersive features and motivational aspects. |
| 2. Do you think the VR training has helped you achieve your learning objectives?  - If so, how exactly?  - Which part helped you the most? | Intrinsic Motivation; Debriefing | Acceptability | Links perceived learning to motivation and post-experience reflection. |
| 3.Would you like to continue the VR training? | Intrinsic Motivation;  Cognitive Load | Demand | Gauges sustained motivation and learner demand. |
| 4. What adjustments would be necessary to make the training optimal for you? | System Usability; Cognitive Load | Practicality | Identifies barriers in usability and cognitive effort relative to feasibility. |
| 5. Did you find the VR training useful?  - If so, what benefits did you feel? | Intrinsic Motivation; Debriefing | Acceptability | Explores perceived value and reflective benefits. |
| 6. Would you recommend this training to your colleagues? | Intrinsic Motivation | Efficacy | Tests external validity of learner motivation as a proxy for programme value. |
| 7. What are the main challenges to the use of VR in medical training? | System Usability; Cognitive Load | Implementation | Identifies barriers to scale-up and sustainable integration. |
| 8. How did you feel during the VR training? | Immersion; Cognitive Load | Acceptability | Captures affective response and cognitive effort within an immersive setting. |
| 9.Did you enjoy it?  - What part did you like most? | Immersion | Acceptability | Pure immersion and enjoyment indicators. |
| 10. In your experience, what might go wrong with this VR training? | System Usability | Practicality; Implementation | Surfaces risk around feasibility and operational barriers. |
| 11. What would you do to make it work better? | System Usability; Debriefing | Practicality | Links design improvements with reflective refinement. |
| 12. Can you think of characteristics of VR technology that can help with engaging with it? | Immersion; System Usability | Practicality | Combines usability with immersive features promoting engagement. |
| 13. Do you have any concerns or safety issues about this VR training? | Cognitive Load; System Usability | Practicality | Safety concerns tied to usability and mental workload. |
| 14. Are there any barriers you can think of that can impact the uptake of this VR training on a larger scale? | System Usability; Intrinsic Motivation | Efficacy; Implementation | Highlights wider adoption issues across learners and systems. |
| 15. Do you have any other questions or issues to raise? | Debriefing | Acceptability | Ensures closure and further reflection. |

**[Mapped interview questions]**

Figures below visually present how each question maps into the domains from the two frameworks. Main questions are presented in grey, the domains from the ITEM framework are presented in blue, and the domains from the Bowen’s framework are presented in red.


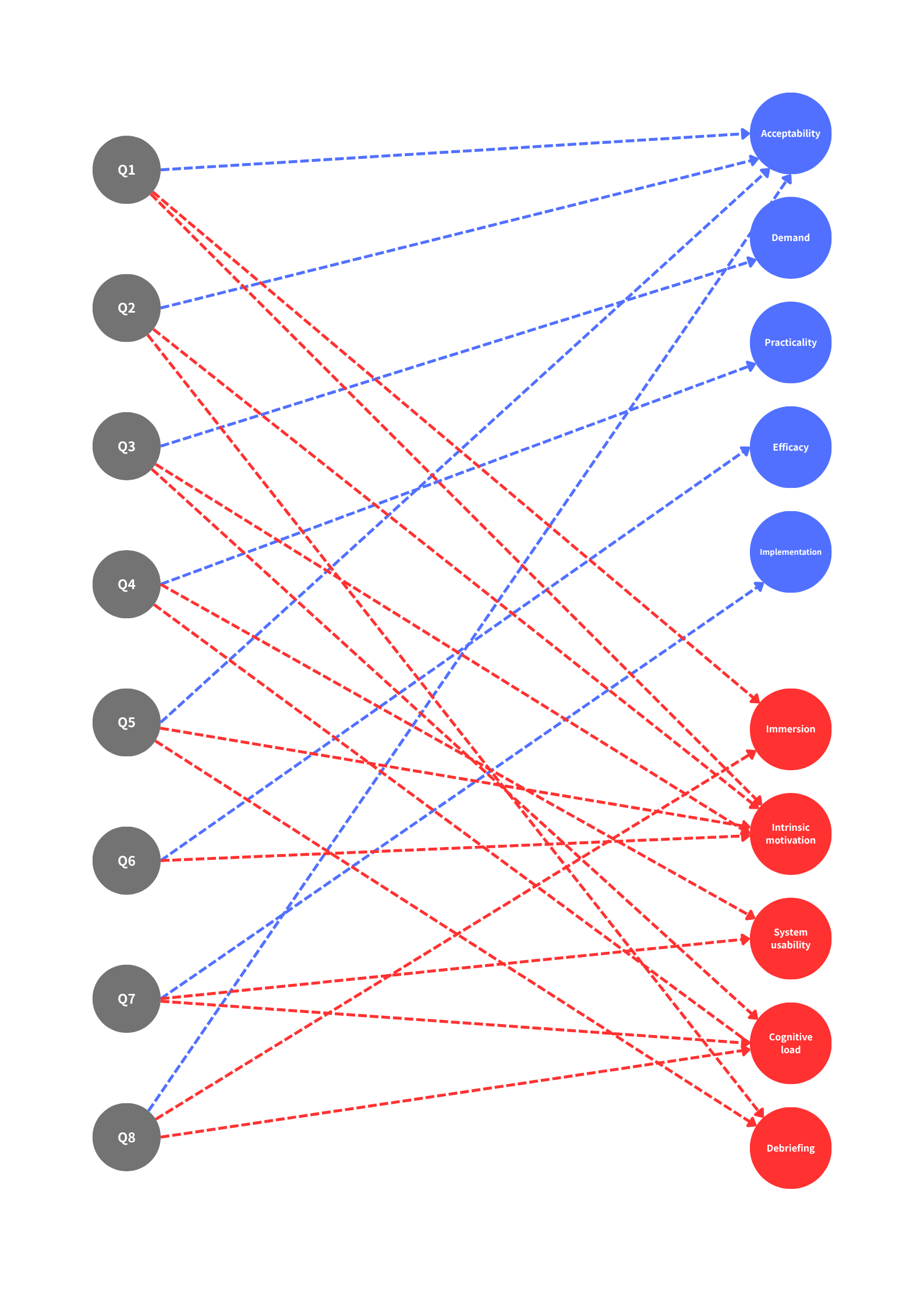


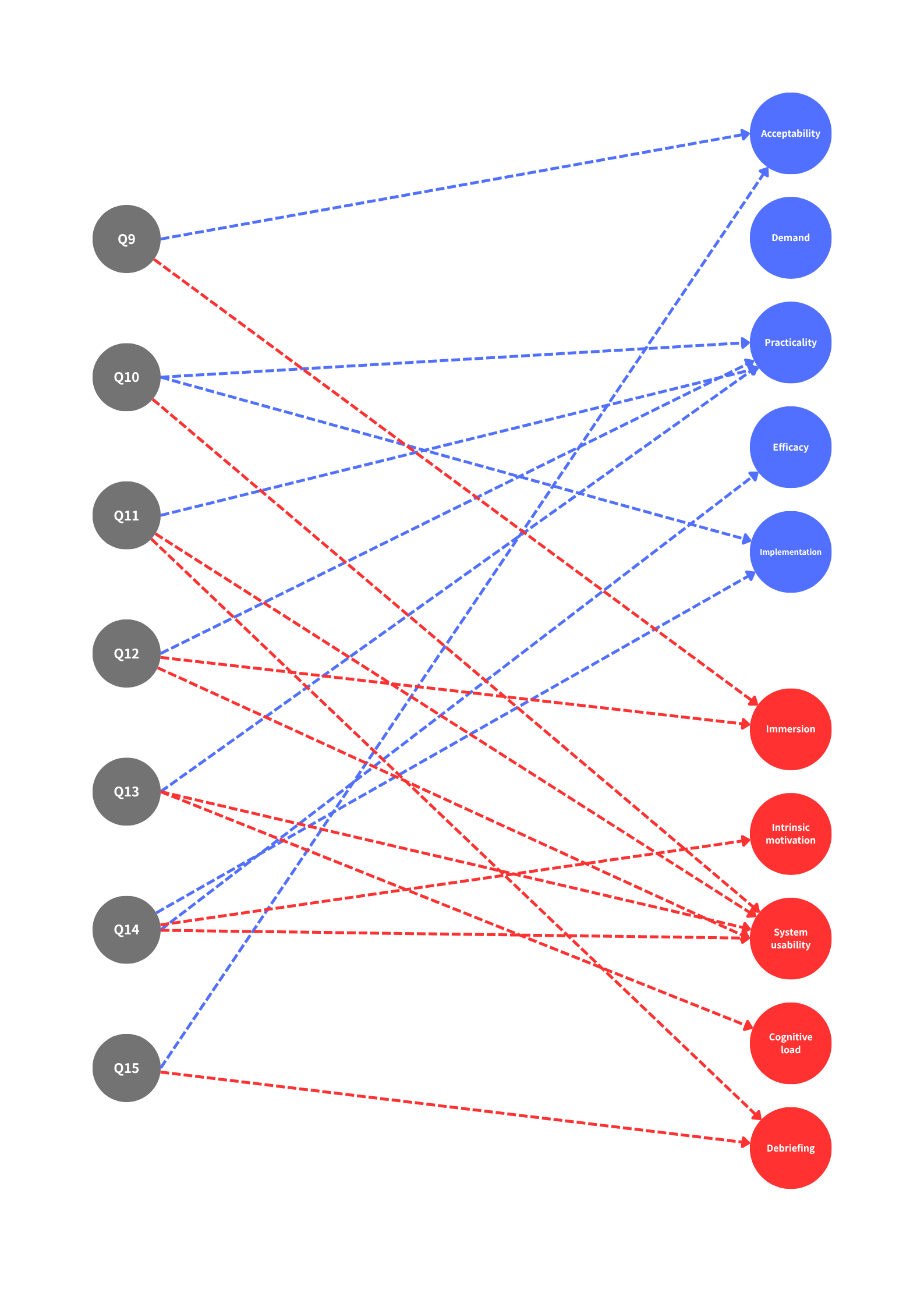

Supplement: Multimedia Appendix 1 [file mededu_v12i1e74301_app1.docx]
